# On Excessive Blood-Letting in Maniacal Affection

**Published:** 1818-11

**Authors:** 


*For the London Medical and Physical Journal.*

### *On excessive Blood-letting in Maniacal Affection;* by Dr. Kinglake.

IT appears to have become too much a prevailing practice to deplenish the sanguiferous system to an indefinite extent in maniacal affection. It has been cursorily and very inadmissibly concluded, that, because persons afflicted with mental derangement exhibit symptoms of high and unyielding excitement, that therefore the powers of life might be safely reduced to an almost unlimited degree of weakness. It would appear that the erroneous hypothesis which has led to this practical delusion has, in numerous instances, been productive of irremediable, and even destructive, mischief. There is no general description of disease incident to the human frame, but includes, in the range of its diversity, certain degrees and states of affection that require a treatment appropriated to its particular character. The practice that might be generally applicable could not be adopted, in especial instances, without manifest impropriety and positive detriment. In maniacal affections in general, there can be no doubt of the eminent benefit resulting from a seasonable and judiciously-limited loss of blood. Vascular distention is, of all stimulating powers, the most universal, and the most efficient, that can obtain in the animal system: to diminish the contents, therefore, of the circulating vessels, is a very direct mode of reducing any inordinate excitement that might be prevailing. In almost every variety of maniacal affection it is conceivable that, from undue dietetic indulgences, unsoothed mental vehemence, extreme watchfulness, interrupted secretions, and an insufficient course of natural evacuations, a degree of vascular fullness may arise, particularly in the brain, that might render the summary remedy of bleeding indispensable. It is possible that the relief afforded by this mode of treatment may be more than temporary, that it may be even lastingly curative. But then the happy result has been produced by a concurrence of favourable circumstances that is inscrutable, and which furnishes no sufficient authority for regarding the loss of blood, or rather the abstraction of stimulus attending its removal, as the efficient cure. In the delirious wanderings attending the progress of febrile irritation, in which no settled derangement of sensorial power has occurred, the exciting cause of the aberration is accidental, and forms no essential part of the diseased action on which the more strict and definite nature of maniacal disease depends. Under such circumstances vascular depletion will often prove availing, not only in overcoming the delirious excitement of the brain, but even of the general irritation constituting the febrile disease. The remedy, therefore, which may be justly considered as suitable and adequate in recent and simple affections of the nervous system, accompanied with symptoms of vascular distention or congestive fullness, cannot be justly held equally efficacious in the more decided and characteristic forms of mania.

Before the crude reasoning, or rather incautious generalization, employed in assimilating all affections of the brain attended with mental derangement, be admitted as a sufficient ground on which to rest the treatment of this description of disease, it should be asked, What is simple delirium, and what is mania?

In examining the diagnostic differences of these several states of disease, it will be seen that the treatment applicable to the one may be contra-indicated in the other; or, at least, may prove not only useless, but highly injurious. In simple delirium, the exciting cause is recent, and the delirious derangement is transient and unestablished: whatever, therefore, may be capable of allaying the hurtful excitement may very naturally suspend and annul the delirium, as the direct effect of such an offensive cause. Bleeding and other evacuations, carried to a due extent, will generally be found efficient means of remedying this state of morbid sensibility. But in maniacal affection, where the sensorial power has been long subjected to the action of diseased influence, where the healthy state of mental excitement has been radically distempered, not a movement is natural, not an impression from the usual exciting causes of rational correctness takes place; all is irregularity and delusion. Whatever may be the exciting cause, the effect is the same. The impression made on the mind stimulates its deranged state: it is, therefore, not likely to recall the power of ratiocination, and to restore lost sanity.

The inveteracy of maniacal disease, then, consists in a vitiated state of nervous sensibility, by which a susceptibility is generated for becoming impressed by natural causes of excitement in a way that is unnatural, and which produces an irrational and deceptious influence. What has induced, and what perpetuates, this distempered state of sensorial power?—Either an inherent or an acquired organic susceptibility for receiving unnatural impressions from natural causes. In either case the disease is untractably obstinate, and forms, as it were, an integrant part of sensibility itself. Is it conceivable, then, that a disease so constituted can have any radical affinity or resemblance to simple delirium, in which the sensorial power is only inordinately excited, but in no respect so vitiated as to be insusceptible of healthy impressions from natural causes? It is hardly credible that a lax scheme of generalizing should have so far assimilated delirium with mania, as to have imagined that both forms of disease would equally give way to profuse and unlimited loss of blood.

This misconception of the true nature of diseases, fundamentally different, though somewhat apparently similar, has really existed, and has authorised an indiscriminating practice that has been always seriously detrimental, and often absolutely destructive, to those who have been the unhappy subjects of it. There are in my reference not fewer than five cases, of recent date and marked character, which are eminently demonstrative of the injurious effects of instituting a plan of treatment for maniacal affection, that might have been, at least, in a degree applicable to simple delirious ailment. In two of the cases alluded to, maniacal disease had occurred, and become characterized by that state of the affection which consists in an inherent organic susceptibility for incorrect impressions from natural causes, in which every sensitive movement was unnatural, and productive of a correspondently disordered state of feeling and reasoning. Lucid intervals occurred of longer or shorter duration, according to the influence of accidental circumstances; but the aberrations were always prompt and irresistible on the slightest occasions. The perception was wildly visionary; the ideas incongruous, neither association nor consistency obtained in their fitful and capricious trains. This disordered state would conjointly furnish a stimulant power, that would render the patients unmanageable, requiring occasional coercion, for the purposes of personal safety and regulation.

Under these unpromising circumstances, much relief had been afforded by moderate bleeding, lax bowels, dietetic abstinence, low temperature, ablution of the head in cold water, mental conciliation carried to the utmost possible extent by avoiding contradiction, by reasonable acquiescence, by anticipating wants, and by promptly gratifying all admissible desires. The intervals of sanity, that were at first not longer than a few hours at a time, in the course of a few weeks were extended by this treatment to three or four days, so as to admit of the patient's going out to visit friends, and even to execute business requiring competent intelligence for its performance. At this period it was suggested that the amended state might be carried the length of perfect recovery by a series of bleeding, that should only be limited by the entire cessation of every symptom of maniacal disease! The curative intention was avowedly founded on placing the system in such a state of insusceptibility for high excitement, as to prevent the possibility of the brain being disturbed in its healthy functions by the ordinary stimulants of life. Abstinence, to the length of almost precluding necessary sustenance, was enjoined, with a view to co-operate with the sedative influence of profuse blood-letting. This practice was pursued, with ardent scientific calculations, and predictions on its beneficial result, but for a short period, when the fatal termination of the cases evinced that there is in bleeding and other debilitating means, as well as in all other things, a *maximum* beyond which it would be unsafe to go. However scientific a scheme of remedy may be, it should be governed by rational experiment. It is rash on all occasions to assume facts; they should occur, and be correctly appreciated, before any authority be inferred from their ideal existence. The removal of vascular distention, and literally emptying the vessels of their contents by immeasurable bleeding, are irreconcilably different: the one is rational, and often curative in its effect; the other is chimerical, and almost necessarily destructive.

The three other cases mentioned were treated according to occurring circumstances. The curative indications that presented were duly regarded. Moderate bleeding, and other suitable evacuations, with the utmost attention to tranquillizing the disordered state of mind, were not omitted.

In maniacal cases of the description of those under consideration, the skilful physician is not more necessary than the sympathising and intelligent metaphysician. Medical aid, with reference to evacuations, diet, and regimen, will be wanting from the former; whilst the latter is as necessary to build up and re-arrange the broken and shattered state of the mind. Ideas, to be intelligently associated in these circumstances, must be scrupulously assorted. Those which will not accord with the prevailing train of thought should be withheld, and those only proposed which would be likely to assimilate and agree with the existing notions of correctness with which the mind may be occupied. If the maniacal patient can be led into a course of reasoning that will tend to correct disorderly impressions, and to evince the delusion by which the mind had been misled, the work of regeneration and recovery will commence, and that too on a foundation that will have its stability in natural feeling and natural conviction. This physic to the distempered mind can only be derived from metaphysical contrivance and management, and will be found of infinitely higher importance in a remedial point of view than any thing than can be attempted by medical aid.

The three patients in question were thus managed; and the event proved its practical value. They all recovered after the lapse of a few weeks; and two of them are now in the full possession of their intellectual faculties, without having had entailed, by their late affection, any sensible injury either on their bodily or mental health. The other is for the most part rational, consistent, and manageable; but still requiring occasional attention to certain circumstances of the maniacal affection that have not so wholly subsided as in the other two instances.

My motive for submitting the above observations to the public is to restrain what would appear to be a very pernicious practice, that of carrying bleeding to a most daring and hazardous extent in affections of the brain, however circumstanced; whether the cases, as before remarked, be founded in simple excitement, as in common delirium, or in distempered and vitiated sensibility, as in mania. To suppose that, because a patient is maniacal, the heart and arteries will endure an indefinite loss of blood, is to imagine a condition of vascular action that has no existence in the animal economy. A given extent of vital action is necessary for the existence of life; if it be sunk below that degree, death must inevitably ensue. The hand that would unwarily, but preposterously, draw blood until vital action be nearly extinguished, with a view to cure a disease, must surely be realizing the medical evil of rendering the remedy worse than the disease. It seems, therefore, that the present licentious abuse of bleeding requires that the experienced and reflecting part of the medical faculty should enter their protest against the immoderate length to which it is often carried.

It has lately occurred to me to see a medical prescription, in which the sanguinary phrase was used of "*Fiat venæsectio ad deliquium!*" Is this more than one remove from *ad mortem?* The moral difference is great and estimable; but the physiological distinction, strictly considered, is but slight and visionary. Who can be assured that the well-intended scientific *ad deliquium* may not prove an accidental *ad mortem?* The very possibility of such a result evinces the censurable flippancy and inconsiderateness of either adopting or carrying such a direction into effect. Were not the works of nature more perfect than those of men, destructive inroads would be often made on them. The deliquium, which it is the avowed object to produce by bleeding, consists in a diminished action of heart, that happily tends to restrain destructive effusion of blood from the sanguiferous vessels. Were not the arterial action mainly dependant on the impulsive contraction of the heart, deliquium from bleeding would be apt to prove mortal; but this wise provision opposes a timely barrier to a fatal waste of the vital fluid. Deliquium from bleeding in the sitting position is a common occurrence, and not accompanied with any serious risk; but that which arises from loss of blood in the recumbent posture is more threatening, and always indicates a diminished state of arterial action that should be rather avoided than designedly incurred. If the deliquium practice in the use of bleeding be ever followed, it should be in the erect, and never in the recumbent, position. The cautious and observant practitioner will be limited in drawing blood rather by the quantity actually taken, than by its effect in inducing deliquium. From six to eighteen ounces should perhaps be regarded as an ample range for safe practice; repeating it, if necessary, as often as the tone and steadiness of arterial action will permit. It sometimes happens that a course of bleeding is pursued as long as symptoms may seem to require it, although a faultering and intermitting action should occur in the pulse. Whenever the heart flags, and becomes irregular in its action under bleeding, it should be considered as a signal for desisting from drawing more, lest a farther loss of blood should induce fatal syncope.

A patient of respectable judgment and seeming veracity was lately under my care, who declared that he had been bled for an affection at the heart to the extent of four quarts at one time, and expressly with a view to occasion fainting. Positive faintness was not induced, but a diminution of vital power that gradually ebbed to an extinction of life in the course of a few weeks, notwithstanding every attempt that was made to sustain and restore the lost strength. This is a solitary instance in my knowledge of so large a quantity of blood being taken away as four quarts: perhaps an equal amount may have been accidentally effused, and life still preserved; but that would be no authority for so rash a practice. It is not uncommon to hear of patients being bled to the extent of from two to three pounds, and that too at short intervals. Certain inflammatory cases, attended with much unyielding tone and energy of arterial action, may require such copious bleedings; but it may be justly presumed that the instances are rare, and that twelve ounces, repeated every six, eight, or twelve hours, according to the urgency of symptoms, would, under any conceivable circumstances of disease, be at once a safer and a more beneficial practice.

*Taunton; September* 15*th,* 1818.

